# Hypokalemia-Induced Rhabdomyolysis as a result of Distal Renal Tubular Acidosis in a Pregnant Woman: A Case Report and Literature Review

**DOI:** 10.1155/2015/947617

**Published:** 2015-12-14

**Authors:** Manasawee Srisuttayasathien

**Affiliations:** Department of Obstetrics and Gynecology, Chaophraya Yommaraj Hospital, Suphan Buri 72000, Thailand

## Abstract

Rhabdomyolysis in pregnancy is a rare occurrence. The manifestation of distal renal tubular acidosis (RTA) for the first time during adulthood is uncommon. According to a review of the literature, pregnancy may predispose individuals to rhabdomyolysis due to hypokalemia. A reduction in interstitial potassium ions could decrease muscular blood flow and lead to muscle injury. This report describes the case of a pregnant woman with rhabdomyolysis induced by hypokalemia resulting from distal RTA. The patient subsequently delivered a healthy newborn.

## 1. Introduction

The frequency of distal renal tubular acidosis (RTA) is low in adult patients. Some studies have reported that pregnancy predisposed women to rhabdomyolysis due to hypokalemia [1, 2]. Two previous studies, including one case report and a single report of four cases, documented pregnant patients whose initial presentation with rhabdomyolysis was secondary to severe hypokalemia [1, 2]. A case report from USA pointed out the need for increasing dose requirements of potassium and bicarbonate in pregnant patients with known distal RTA to maintain the electrolyte balance [3]. A clinical investigation of the mechanism of rhabdomyolysis in potassium depletion revealed that potassium released from contracting skeletal muscle cells into interstitial fluid directly dilated adjoining arterioles, mediating the potassium-induced increase in muscular blood flow. The failure to release potassium during skeletal muscle contraction leads to diminished hyperemia, potentially resulting in muscle injury, ischemia, and necrosis. Potassium-depleted animals showed elevated creatine phosphokinase (CPK) activity in serum, suggesting a loss of skeletal muscle integrity [4]. This paper reports a case of a third-trimester healthy pregnant woman who presented with proximal muscle weakness, increased CPK, and severe hypokalemia. After recovery, she delivered a normal newborn.

## 2. Case Report

A 27-year-old woman, living in the central region of Thailand, gravida 1, presented at 37 weeks and 5 days' gestation with complaints of both arm and leg weakness and painful muscle cramps. Her symptoms had begun 5 days earlier. She had myalgia and showed progressive difficulty of proximal muscle activities more than distal muscle activities, without signs of muscle wasting. The patient had no ptosis, and her respiration was normal. A dermatologic exam was unremarkable. She had no history of diarrhea or toxin exposure. Her daily medication consisted of a tablet of ferrous fumarate and a single supplement containing iron, iodine, and folic acid (Triferdine). She had no significant prior antenatal history, no hearing problem, no sign of autoimmune disease, no history of dry eyes or dry mouth, no history of diuretic intake, no history of chronic alcohol intake, and no family history of weakness and hypokalemia.

In the initial assessment, the patient was afebrile, and her blood pressure was 109/65 mmHg. A neurological examination revealed the following motor power: grade 3/5 in both arms, grade 4/5 in both forearms, grade 4/5 in both thighs, and grade 5/5 in both legs. All the deep tendon reflexes were 2^+^. The well-being of the fetus was assured by a nonstress test. Normal fetal bone development and mineralization were observed by ultrasound.

Investigations showed a total leukocyte count of 8,310/mm^3^ (5000–10000), with a neutrophil count of 68% (40–75), a lymphocyte count of 24% (20–50), a monocyte count of 6% (2–10), and eosinophil count of 2% (1–6). The hemoglobin was 11.7 g (11–15), platelets were 261,000 cells/mm^3^ (140,000–400,000), serum potassium was 2.0 mmol/L (3.5–5.0), sodium was 139.5 mmol/L (135–145), and chloride was 108 mmol/L (97–110). Bicarbonate was 19.9 mmol/L (22–32), and it decreased over the next few days to 16.0 mmol/L. In addition, albumin was 3.2 g/dL (3.4–5.0), calcium was 9.7 mg/dL (8.4–10.4), corrected serum calcium was 10.34 mg/dL, magnesium was 1.8 mg/dL (1.8–2.4), phosphorus was 3.8 mg/dL (2.5–4.5), blood sugar was 114 mg%, BUN was 4.1 mg/dL (7–18), creatinine was 0.52 mg/dL (0.55–1.02), aspartate aminotransferase (AST) was 194 U/L (15–37), alanine aminotransferase (ALT) was 124 U/L (0–65), globulin was 2.7 g/dL (2.8–3.3), total bilirubin was 0.43 g/dL (0.0–10.0), direct bilirubin was 0.22 mg/dL (0–0.50), and alkaline phosphatase was 137 U/L (40–150). Urine analysis was negative for blood and urinary tract infections. The urinary pH was 7.0 (5.0–7.5) on a pH meter, and the urinary ketone level was 2^+^. CPK was 5,338 IU/L (<145). Thyroid function tests were in the normal limit for a third-trimester pregnancy, and serum morning cortisol was mildly increased. Urine was collected after 24 h. The 24 h urine potassium level was elevated at 77 mmol, suggesting hypokalemia from renal losses. Serum anion gap was 13.6 mmol/L. The reduction in bicarbonate and normal anion gap pointed to normal anion gap metabolic acidosis. The patient had no history of extrarenal causes of normal anion gap metabolic acidosis such as vomiting, diarrhea, gastrointestinal fistula, and laxative abuse. These data suggest renal tubular acidosis as a cause. Urine pH of 7.0 pointed out that kidneys could not acidify urine compatible with distal tubular dysfunction. Her serum phosphate was normal and she had no glucosuria. Serum bicarbonate easily increased to 20.9 mmol/L after 0.36 mEq/kg administration. So, all data suggest that she had distal RTA. Then she had no history and no clinical suggestion of anemia and autoimmune disease and had no history of deafness, and besides the fact that screening tests for the underlying causes of the distal RTA were normal the etiology in this case was indeterminate. The serum uric acid was 4.1 mg/dL, antinuclear antibody was negative, and a whole abdomen ultrasound showed mild hydronephrosis, without any detectable renal mass or stones.

Treatments included hydration, correction of the metabolic acidosis with alkali therapy, and potassium supplementation. The patient's clinical and laboratory test results gradually improved. One week after starting the treatments, all the laboratory test results were negative. The patient was discharged.

At 41^2/7^ weeks of gestation, the patient was diagnosed with failure to progress and was taken to the operating room for a cesarean section. A healthy male newborn was delivered. The newborn weighted 3,360 g at birth, and he had an Apgar score of 9 and 10 at 1 and 5 min, respectively. Thin meconium-stained amniotic fluid was found. The operation and postpartum period were uneventful. On follow-up, serum electrolytes, CPK, and creatinine were within normal limits. At the 6-week postpartum check-up, the patient was in good health. She received an injection of depot medroxyprogesterone acetate (DMPA) for contraception.

## 3. Discussion

Severe hypokalemia, defined as serum potassium under 2.5 mmol/L, can cause many signs and symptoms, such as fatigue, nausea, vomiting, muscle weakness, ileus, and eventually rhabdomyolysis. Morbidity and mortality are associated with unrecognized hypokalemia and can lead to respiratory failure and death [5]. The pregnant woman was diagnosed with hypokalemia due to primary distal RTA, which is uncommon in adults. The etiology of distal RTA in this case is not well understood. One report suggested that RTA could be transient in pregnant patients and that they recovered after delivery [6]. However, another report found that pregnancy could worsen RTA [3]. Therefore, pregnant patients with RTA should undergo regular check-ups of kidney function and electrolytes.

Rhabdomyolysis is a potentially life-threatening syndrome resulting from the breakdown of skeletal muscle fibers, with leakage of muscle contents into the circulation [7]. Severe hypokalemia can lead to rhabdomyolysis due to the diminished blood flow of muscle arterioles. An increased predilection for hypokalemia-induced rhabdomyolysis has been reported in pregnancy, with unknown pathophysiology [1]. In the present case, the patient's laboratory tests revealed increases in CPK, AST, and ALT from muscle injury. These decreased shortly after treatment, with no further complications.

Severe acute metabolic acidosis in pregnancy can impair the fetal circulation, causing fetal distress. However, the acidosis in the present case was detected early, and the patient was treated with alkali therapy and closely monitored. The well-being of the fetus was reassured by NST and an ultrasound while the patient was hospitalized, and the delivery was uneventful. Theoretically, chronic RTA can prolong maternal metabolic acidosis, impairing fetal growth and development and causing fetal distress [3]. Fortunately, this did not occur in the present case. Therefore, it is likely that the RTA in the current case was of short duration.

As DMPA has no metabolic effect on the electrolyte balance, it can be safely used as a contraceptive. A previous report described recurrent episodes of rhabdomyolysis due to uncorrected hypokalemia in a patient with asymptomatic distal RTA during two consecutive pregnancies [8]. Thus, if the patient in the current case desires more children, early antenatal care and electrolyte monitoring should be considered. Some cases of type 1 RTA were reported to be inherited in an autosomal dominant pattern [3]. Monitoring the growth and development records of these children may shed light on the potential effects of RTA.
